# Production of Hybrid Joints by Selective Laser Melting of Maraging Tool Steel 1.2709 on Conventionally Produced Parts of the Same Steel

**DOI:** 10.3390/ma14092105

**Published:** 2021-04-21

**Authors:** Ludmila Kučerová, Ivana Zetková, Štěpán Jeníček, Karolína Burdová

**Affiliations:** Regional Technological Institute, Faculty of Mechanical Engineering, University of West Bohemia, Univerzitni 8, 30614 Plzen, Czech Republic; zetkova@rti.zcu.cz (I.Z.); jeniceks@rti.zcu.cz (Š.J.); kburdova@rti.zcu.cz (K.B.)

**Keywords:** additive manufacturing, maraging tool steel, hybrid joints, powder bed fusion

## Abstract

Joining additively manufactured (AM) complex shaped parts to larger conventionally produced parts can lead to innovative product designs. Another alternative is direct deposition on a conventional semi-product. Therefore, similar joints of maraging tool steel 1.2709 were produced by AM deposition of powder of this steel on a bulk conventionally manufactured steel part. The resulting hybrid parts were solution annealed and precipitation hardened. Solution annealing at 820 °C for 20 min was followed by furnace cooling. Precipitation hardening was performed at 490 °C for 6 h. The mechanical properties of the samples were characterised using tensile testing and hardness measurement across the joint. Metallographic analysis was also carried out. The tensile properties of the AM and conventionally produced steel after equivalent heat treatments were also determined as the reference values. The mechanical properties of the hybrid parts are close to the properties of both steels. The hybrid parts in the as-built condition had a tensile strength of 1029 MPa and a total elongation of 14%. Solution annealing did not change these properties significantly, except for yield strength, which decreased by approximately 150 MPa. After precipitation annealing, the strength was higher, 2011 MPa, and total elongation dropped to 5%.

## 1. Introduction

Additive manufacturing (AM) is a rapidly developing family of techniques for the chipless production of complex shaped objects. There are several recently developed additive technologies enabling the processing of a wide range of materials [1,2,3,4]. This work will focus on a powder bed fusion technology of selective laser melting (SLM). The SLM process is for the printing machines produced by ESO GmbH also called direct metal laser sintering (DMLS) and it uses the controlled movement of a laser beam to melt layers of powder in the desired areas [5]. This technology has many advantages, such as nearly wasteless production due to the recycling of most of the unmelted powder. Drawbacks include the long production times, which are necessary for the layer-by-layer building process. Considering the limited chamber sizes of commercially available printers and the high cost of AM products, there is a good reason for attempting to join these AM special parts to larger conventionally produced semi-finished products.

Apart from using various welding methods, direct powder deposition (DPD) appears to be a viable option for producing hybrid components which combine conventionally manufactured and AM parts. Powder bed technologies are traditionally not considered suitable for producing similar or dissimilar joints with conventionally manufactured materials. Even though the DMLS powder bed process offers better precision of deposition than DPD, powder bed methods have significant limitations in the shape of the conventionally manufactured substrate, as the first layer needs to be deposited on a completely flat surface. Therefore, very little research has been published dealing with hybrid parts or bimetals created by DLMS deposition of maraging steel on conventionally manufactured steel substrates [6,7,8,9], and still less about the effect of post-processing heat treatment on these hybrid parts. In several cases [6,7], the deposition was only partially successful, as the parts failed within the joint area during subsequent mechanical testing. This implies that there is a need for further study in this field, as successful deposition of metals on conventionally manufactured semi-finished products could become a new method for making hybrid parts, contributing to the integration of AM into conventional production chains [10,11,12,13]. The possible application of those hybrid parts could be for example in hybrid tooling or precision repairing, as DLMS of the maraging steel is increasingly used in the production of conformal cooling of shaped inserts for non-ferrous metal castings or for the injection molds. In both cases, the higher precision of DMLS could be an advantage. Although the necessity to deposit the first layer on a flat surface might be a disadvantage for DMLS in the repairment processes, there are also many instances where this factor does not play any role. For example, in the molds, the most critically loaded part is the transition area of the bulker base material into the specific, often thinner part with more complex geometry where the cracking quite often occurs. This exposed part is typical for mold inserts or sliders. In those cases, the repair work has to start with cutting off the special part and its complete rebuild on the flat base material. This makes DLMS methods as suitable as DPD in the terms of flexibility while keeping it a more suitable choice for the precise replication of the original inner geometry, particularly for smaller objects with more complicated shapes.

In this work, maraging steel 1.2709 powder was deposited on conventionally manufactured parts of the same steel using SLM technology. A previously published work using the same materials was one of those which reported the failure of the hybrid sample within the joint [7]. The aim of this work was to obtain a hybrid part where the joint area would not necessarily present the weakest point and the application of post-processing heat treatment would optimise the properties of the hybrid part and bring them at least to the level of the properties of a conventionally produced maraging steel. Maraging steel 1.2709 is frequently used in AM due to its good weldability and the possibility to adjust its final mechanical properties by post-processing heat treatments to values ranging from 1000 to 2000 MPa tensile strength and 4% to 14% elongation [14,15,16,17,18]. This steel possesses a relatively soft matrix of extremely low carbon martensite which can be strengthened during precipitation hardening treatment by the precipitation of intermetallics [19,20,21,22]. The mechanical properties of this steel could be further tailored by the presence of a controlled fraction of retained or reversed austenite, as some researchers suggest the possibility of triggering the TRIP (transformation induced plasticity) effect in this steel [23]. The steel is used in tooling, aerospace and automotive industries [24] with safe long-term operation ensured by the stability of its microstructure and properties up to temperatures around 500 °C. Its potential for use in future applications in the automotive industry sparked the idea of joining additively and conventionally manufactured parts and investigating resistance spot welding of AM maraging steel [25].

## 2. Materials and Methods

### 2.1. Materials

Maraging tool steel 1.2709 was used in the form of powder sold by the company ESO GmbH (Krailling, Germany) as MS1, and also in the form of conventionally produced VACO 180 steel (Bohdan Bolzano, s.r.o., Kladno, Czech Republic). It is also known as maraging steel 18Ni300 grade or X3NiCoMoTi 18-9-5 (Table 1).

The powder is produced by gas atomization, which leads to relatively spherical particles. The conventionally manufactured maraging steel VACO 180 was supplied by Bohdan Bolzano s.r.o. in the form of hot rolled and solution annealed (820 °C/1 h, air cooled) slabs with a 100 mm × 20 mm cross-section and 500 mm long. Cylindrical samples were manufactured from the slabs as the substrate for subsequent deposition with the axis parallel to the rolling direction, a diameter of 10 mm and a length of 45 mm. The conventionally manufactured steel contained a microstructure of lath martensite.

### 2.2. Additive Manufacturing

An EOS M290 3D printer (ESO GmbH, Krailling, Germany) was used for additive manufacturing of the maraging steel using the standard parameters recommended by EOS GmbH (Table 2). A stripes hatch strategy was used for additive manufacturing with a hatch spacing of 110 μm. The stripe width was 10 mm (default EOS M290 Direct Part setting) with the stripes overlap of 0.05 mm (also default EOS M290 setting). The orientation of the laser tracks between the two following layers changed by 67°. The thickness of printed layers reached 40 μm. The lower skin layer was not produced in this case, as the first layers were deposited directly on the conventionally produced maraging steel cylinder. Nevertheless, the first layer was melted twice to ensure a high-quality joint between the conventionally produced and the AM maraging steels. The baseplate was pre-heated to 40 °C and this temperature was maintained during the AM process. Nitrogen gas was used as a protective atmosphere during AM, and the builds were heat-treated in a protective argon atmosphere.

**Figure 1:**
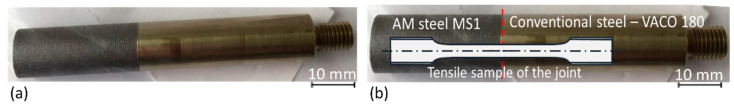
(**a**) Hybrid part (MS1 left, VACO 180 right), (**b**) position of the tensile test sample for determining the mechanical properties of the hybrid part.

To keep the accuracy of the final cylindrical shape of the hybrid part, centering pivots were used to ensure the exact position of the base plate in the machine. Threaded holes were made in the base plate for this purpose. The cylinders prepared from the solution annealed slabs of conventional maraging steel VACO 180 were provided with threads at one end (Figure 1) for precise mounting onto the base plate.

**Figure 2:**
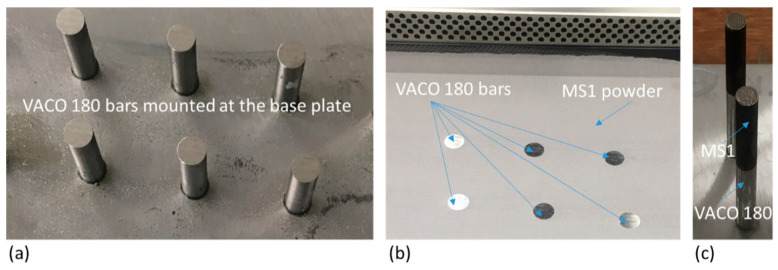
Additive manufacturing process: (**a**) VACO 180 bars mounted at the base plate after grinding; (**b**) printing chamber filled by MS1 powder up to the top bases of VACO 180 bars ready for additive manufacturing; (**c**) finished parts (still mounted at the base plate).

The height differences between the mounted cylinders were hundredths of a millimetre, which is not acceptable for subsequent AM. Therefore, the whole assembly of the base plate with the cylindrical samples of conventional steel VACO 180 was ground to create a common plane containing all the top faces of the cylinders, which was then oriented parallel to the recoater (Figure 2). This rather time-consuming preparation was necessary to ensure the precise build of the hybrid part. Details of the building process and images of the semi-products in various production stages were previously given in [6]. MS1 powder was deposited to produce the cylindrical portion of the sample with the same diameter of 10 mm and a length of 30 mm, making the total length of the hybrid parts 75 mm (Figure 1). The AM was carried out with the axis of the cylinder parallel to the building direction.

A set of the nine cylinders was additively manufactured in a standard way at a separate base plate, using the same powder, sample orientation and printing parameters as in the case of the hybrid parts to produce samples for mechanical testing of AM steel in various conditions (without heat treatment, solution annealed and precipitation hardened).

### 2.3. Heat Treatment 

**Figure 3:**
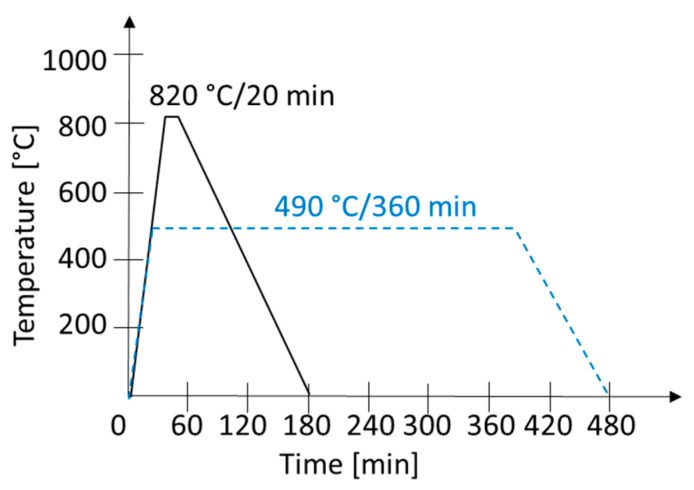
Post-processing heat treatments. The solid line shows the solution annealing schedule, the dashed line shows the precipitation hardening schedule.

One of the issues in DMLS additive manufacturing is the high residual stress in the build due to the steep heating and cooling gradients. To prevent undesirable distortion or even cracking of the builds, subsequent heat treatment is recommended, particularly for larger or more intricate products. To evaluate the effect of this post-processing heat treatment on the microstructure and the mechanical properties of the MS1/VACO 180 parts, some parts were kept in the as-built state, while others underwent two types of post-processing heat treatment. Four hybrid parts were produced for each heat treatment. Three parts were used for tensile testing of the hybrid joint and the last one was used for metallography and hardness measurements. Six separately printed AM cylinders and six parts of VACO 180 were also heat-treated in the same way to produce reference tensile test samples of individual steels (three parts per material and processing conditions). The first heat treatment was precipitation annealing with a 6 h hold at 490 °C. This treatment is used for intensive strengthening of maraging steel by the precipitation of very fine intermetallic particles which are homogeneously dispersed in the soft matrix. The second heat treatment was solution annealing at 820 °C with a 20 min hold and subsequent cooling to room temperature (Figure 3). The main aim of this treatment is to homogenise the microstructure and to reveal the residual stresses in the AM steel, which might otherwise cause distortion or cracking of the AM parts.

### 2.4. Characterisation

The virgin MS1 powder was investigated in a scanning electron microscope (SEM) to determine the shape, size and size distribution of the particles. The average powder particle size was established by image analysis of SEM images at a magnification of 1000×. Three different random images with a total of 250 particles were measured. The microstructure of the powder was analysed on a metallographic section using an SEM with energy-dispersive X-ray spectroscopy (EDS) and electron back-scattered diffraction (EBSD) detectors. The hybrid samples were cut along the rotation axis and the central part with the joint was used for the metallographic sections, i.e., the side cross-section was observed in the AM part. Longitudinal sections were also prepared from the fractured samples after tensile testing to find the location of the fracture with respect to the joint. All metallographic sections of the powder and the steels were prepared in a conventional way by hot mounting (CitoPress-15, Struers GmbH, Ballerup, Denmark) in a conductive resin (PolyFast, Struers GmbH, Ballerup, Denmark), grinding and polishing with 3 and 1 μm diamond suspensions. Final polishing in colloidal silica was used for EBSD analysis. The microstructures of both steels, their interfaces and the powder were observed using a Zeiss EVO 25 scanning electron microscope with a LaB6 cathode (Zeiss, Oberkochen, Germany), a Zeiss Crossbeam microscope with a FEG cathode (Zeiss, Oberkochen, Germany) and a BX61 Olympus light microscope (Olympus, Shinjuku, Tokyo, Japan). All the samples were etched with 3% Nital (Lach-Ner, s.r.o., Neratovice, Czech Republic) with the exception of the solution-annealed samples, which were etched with dilute aqua regia (H_2_O/HNO_3_/HCl = 6:1:3, (Lach-Ner, s.r.o., Neratovice, Czech Republic) for SEM observation.

The phase analysis (austenite and ferrite contents) was carried out by EBSD analysis and by X-ray diffraction phase analysis (XRD) using an AXS Bruker D8 Discover diffractometer with a Co source (Bruker, Billerica, MA, USA). XRD was used for phase quantification, while the main purpose of the EBSD analysis was to determine the morphology and distribution of the retained austenite. XRD spectra were evaluated within the range of 25° to 110° and the austenite fraction was determined from intensities of (111), (002) and (022) peaks.

Hardness HV 10 was measured using a Wollpert 432-SVD (Wilson Instruments, Norwood, MA, USA) to determine the mechanical properties of the additively manufactured and conventional maraging steels in various conditions. The average values of five measurements per sample are provided. Line measurement of microhardness HV 0.1 and microhardness mapping were done using a Leco LM 248 AT (Leco Corporation, St. Joseph, MI, USA) to characterise the continuously changing local mechanical properties at the interfaces of both materials. The linear measurement was carried out at a distance of 2 mm across the interface. The interface was always placed in the centre of the measured area. The spacing of the imprints was 0.1 mm for line measurement and 0.05 mm for hardness mapping. The hardness mapping was performed with an imprint spacing of 0.05 mm and 100 g load (HV 0.1), using 288 imprints in total covering an area of 1.7 mm × 1 mm. A dwell time of 10 s was used for all hardness and microhardness measurements.

Samples with cylindrical bodies were machined for mechanical testing, with a diameter of 4 mm, gauge length of 20 mm and an M8 thread head. Rough and finish turning were applied to achieve the surface roughens of about 1.6 Ra. The central portion of the tensile sample was located at the interface of the conventional material and the build (Figure 1) in the case of hybrid parts. The mechanical properties of the AM and conventional maraging steels after heat treatments and without heat treatment were also obtained using the same sample geometry as in the case of hybrid parts to analyse the influence of the steel properties on the behaviour of the hybrid parts. The tensile properties of the AM steel were determined in the z-axis (build direction perpendicular to the platform), to enable direct comparison with the hybrid part, which was also built along the z-axis. Three samples were tested at room temperature for each combination of processing parameters, and the average mechanical properties, including standard deviations, were evaluated. Tensile testing was done using a Zwick Roller Z250 testing machine (ZwickRoell, Ulm, Germany), according to EN ISO 6892-1 [26], with a strain rate of 0.0067 s^−1^.

## 3. Results and Discussion

### 3.1. Microstructure Analysis of the Powder

**Figure 4:**
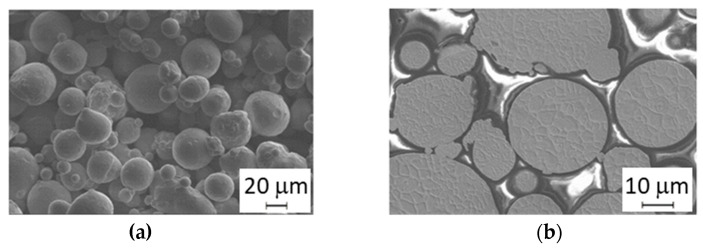
Scanning electron microscopy (SEM) images of powder MS1: (**a**) shape of powder grains; (**b**) cross-section showing cellular microstructure.

**Figure 5:**
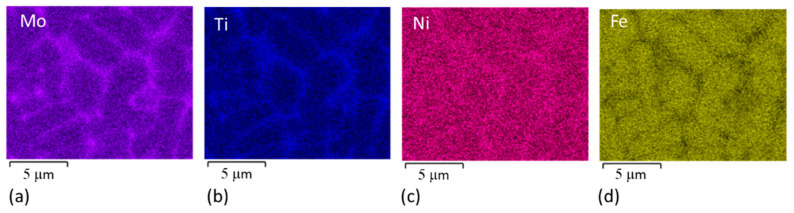
Energy-dispersive X-ray spectroscopy (EDS) analysis on a metallographic section through the powder with distinct segregation of (**a**) Mo, (**b**) Ti, (**c**) slight segregation of Ni and (**d**) lack of Fe along cell boundaries.

**Figure 6:**
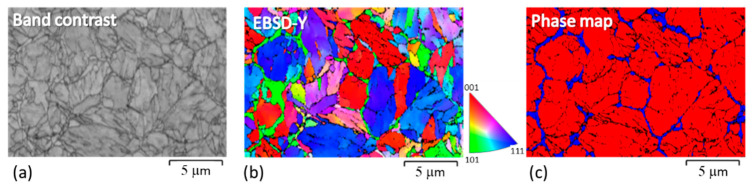
Electron back scattered diffraction (EBSD) analysis of the metallographic section through the powder: (**a**) band contrast; (**b**) Inverse pole figure in the direction Y (IPF–Y figure); (**c**) phase map with blue—retained austenite at cell boundaries, red—martensitic matrix.

The shape and size of the virgin powder were determined by SEM observation and image analysis. The average particle size was 25 μm. The particles had quite regular spherical shapes (Figure 4a). Details of the powder are given in [27]. Some satellites (very fine spherical particles attached to the surfaces of larger ones) were found. The microstructure of the grains was cellular (Figure 4b), consisting of a mixture of finer and coarser cells. The cell microstructure corresponds closely to the distribution of the alloying elements. Strong segregation was found, with Ti and Mo predominantly at the cell boundaries, and there was also a slightly increased Ni content at the cell boundaries (Figure 5). EBSD analysis revealed that the matrix microstructure was lath martensite with thin films of retained austenite along the cell boundaries (Figure 6).

Comparison of the EDS and EBSD maps taken at the same magnification clearly demonstrates that the increased content of the alloying elements at the cell boundaries contributed to the stabilisation of the retained austenite in these areas.

### 3.2. Microstructure Analysis of Hybrid Parts

**Figure 7:**
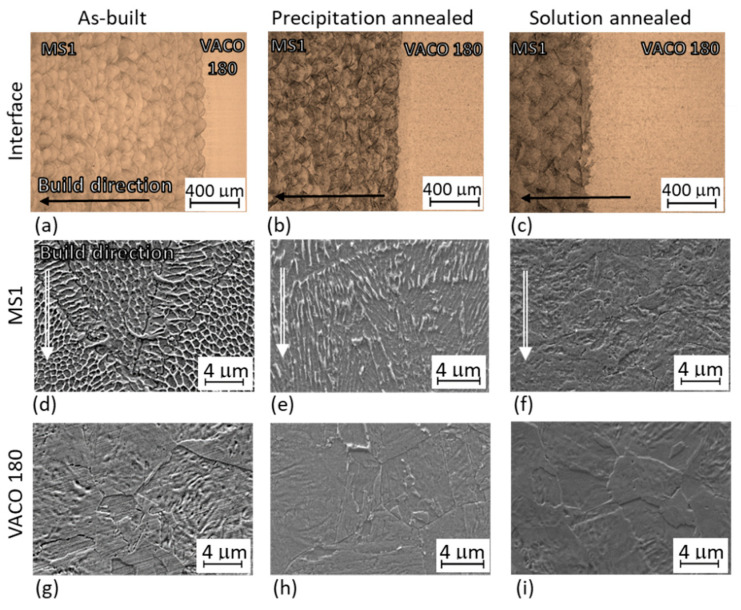
Optical micrographs of the interface: (**a**–**c**); SEM Micrographs of additively manufactured MS1 steel (**d**–**f**); the microstructure of VACO 180 (**g**–**i**). Hybrid parts without post-processing heat treatment (**a**,**d**,**g**); with precipitation hardening (**b**,**e**,**h**); with solution annealing (**c**,**f**,**i**).

**Figure 8:**
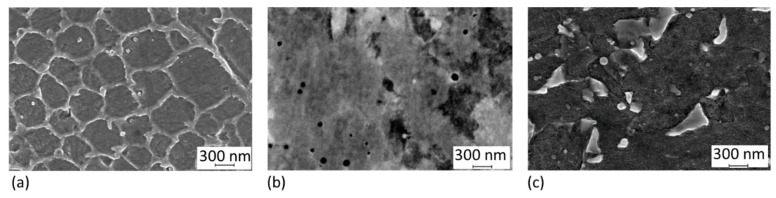
Detail SEM images of MS1 steel in: (**a**) as-built condition etched, observed in secondary electrons; (**b**) as-built condition polished, observed in back-scattered electrons; (**c**) precipitation hardened condition.

**Figure 9:**
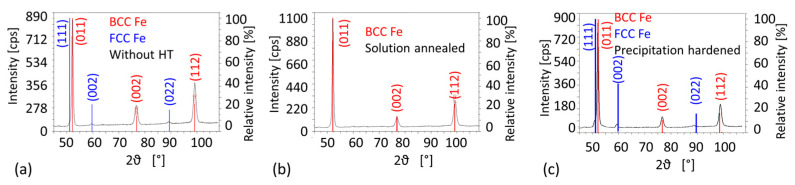
X-ray diffraction (XRD) spectra of additively manufactured (AM) steel: (**a**) without heat treatment (HT); (**b**) solution annealed; (**c**) precipitation hardened. Body centered cubic lattice of ferrite (BCC Fe), Face centered cubic lattice of austenite (FCC Fe).

**Figure 10:**
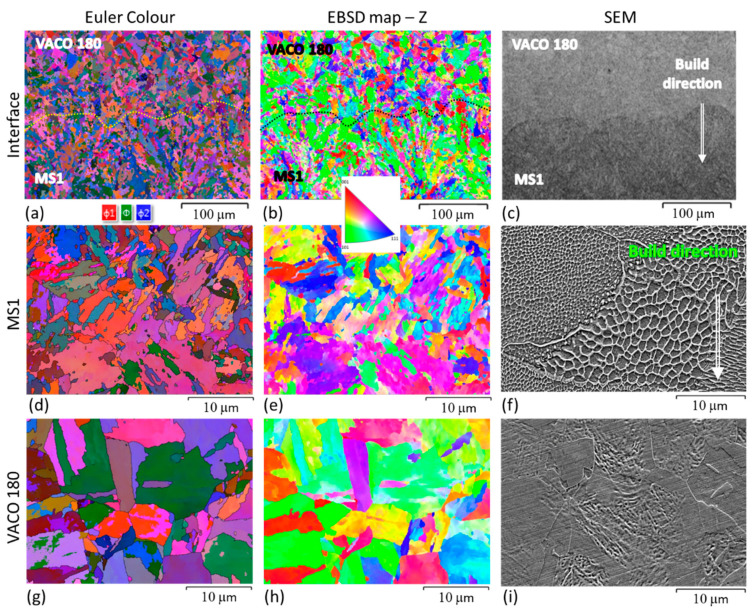
Hybrid part without post-processing heat treatment: Euler maps (**a**,**d**,**g**); Inverse pole figures in Z direction (IPF–Z direction) (**b**,**e**,**h**); and SEM micrographs (**c**,**f**,**i**); VACO 180-MS1 Interface—the interface is marked with a dotted line in the EBSD images (**a**,**b**,**c**); additively manufactured MS1 (**d**,**e**,**f**); conventionally prepared VACO 180 (**g**,**h**,**i**).

The microstructure of the AM steel was observed in a longitudinal cross-section. Traces of sintered powder layers were revealed as “lines” of half-ellipse shaped melt pools (Figure 7). Due to their distinctive shape, they are sometimes also called “fish scales” [14]. This microstructure is completely different from that of conventional steel with the same chemical composition. The conventional maraging steel contained laths of soft martensite where the solid solution was super-saturated with substitutional alloying elements (Figure 7g). The additively manufactured steel possessed a significantly finer cellular microstructure, with a martensitic matrix and around three percent of retained austenite along the cell boundaries (Figure 7, Figure 8a, Figure 9a and Figure 10a–f). The location of the retained austenite was connected with an increased concentration of alloying elements along the cell boundaries, which is assumed to result from microsegregation during solidification when alloying elements preferentially partition into the liquid phase [15,22]. This effect is also clearly seen in the microstructure and distribution of the alloying elements in the initial powder (Figure 5 and Figure 6). Particles of various sizes with increased Ti content were observed in the AM microstructure (Figure 8a,b), and they are expected to correspond mainly to Ni_3_Ti particles which have been reported by other authors to be dominant precipitates in AM maraging steel [21,22] and Ti oxides [28]. Precipitation and epitaxial growth of cells across the boundaries of laser tracks (Figure 7d) are caused by intrinsic heat treatment occurring during additive manufacturing. Due to the incremental nature of the AM process, the deposited layers are repeatedly heat-treated during melting and solidification of the layers above.

According to XRD measurements (Figure 9), the AM steel contained about 3% of retained austenite in as-built condition without heat treatment (Figure 9a). No austenite was found in the conventional VACO 180 steel in an initial condition. This is in agreement with other research, as solution annealed conventional maraging steel does not typically possess any austenite in the microstructure, while several percent of retained austenite is commonly present in AM steels in the as-built condition, as reported by [15,22,28,29,30]. The austenite phase fraction detected by those authors in the AM as-built maraging steel 1.2709 varied from approximately 3% to 6%.

In the hybrid sample without post-processing heat treatment (Figure 10a–i), EBSD revealed that the AM microstructure is finer (Figure 10d,e) than that found in the conventional steel (Figure 10g,h). This can be seen in the EBSD image of the interface region (Figure 10a,b), where the very fine AM steel (MS1) is at the bottom, with subgrains forming a fan-like morphology within individual laser tracks. At the interface, it transitions into randomly distributed fine grains in the heat-affected zone of the conventional VACO 180 steel. This grain refinement was caused by rapid heating and cooling of the VACO 180 surface layer during the deposition of the first layers of MS1 steel.

Slight coarsening of these grains is already apparent at the very top of the images (Figure 10a,b). This is in agreement with optical microscopic observations at higher magnification (Figure 7c). The micrograph shows the thin heat affected layer of the conventional steel along the interface. This layer is from 120 to 195 μm thick.

The precipitation hardening of the hybrid part at 490 °C resulted in the precipitation of a dispersion of very fine newly-formed particles in the maraging steel. However, the particles are visible only at high magnifications, as was previously described by our group [14]. Laser tracks are still discernible in the microstructure, and prior austenite grain boundaries can be found in the light micrographs (Figure 7b,e). Even though the microstructure of the AM steel still retained some traces of laser processing, the original cell microstructure was partially dissolved. The austenite content increased to about 9% (Figure 9c) with the formation of the first islands of reversed austenite during the hold at 490 °C. This reversed austenite was found predominantly along the prior cell boundaries (the thin white films in the micrographs in Figure 7e and Figure 8b). On the other hand, VACO 180 only contained approximately 3% of retained austenite after precipitation hardening. Since no austenite was detected in the as-received bars, the reversion of a small amount of martensite to austenite during precipitation hardening can be expected. However, it should be noted that 3% is close to the detection limit of our X-ray diffraction analysis, which means that austenite contents slightly below 3% might be missed by this method even if present. The difference between the austenite content in the AM and conventional steels could be nevertheless attributed to the uneven distribution of the alloying elements caused by rapid heating and cooling during the AM process [15,30]. This non-uniformity promoted the retention of 3% austenite in the as-built microstructure and supported the reversion of another 6% of austenite during precipitation annealing of the AM steel. The heat-affected layer on the conventional steel side of the interface is visible in the light micrographs. The thickness of this layer is no more than 90 to 120 μm (Figure 7b,e).

**Figure 11:**
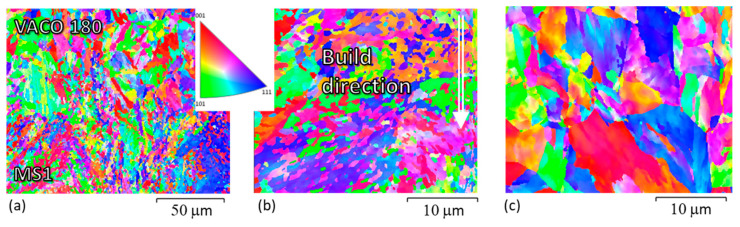
EBSD (IPF–Z direction) of a hybrid part annealed at 820 °C: (**a**) VACO 180-MS1 interface; (**b**) additively manufactured MS1; (**c**) conventionally prepared VACO 180 steel.

The solution annealing temperature of 820 °C is above the recrystallisation temperature of this steel and in the fully austenitic region. Therefore, the original cells of the AM steel dissolved completely (Figure 7f), resulting in a more uniform distribution of the alloying elements. However, even after solution annealing, the microstructure still retains some features of the as-built steel, and the laser tracks remain visible (Figure 7c). No retained austenite was detected by X-ray diffraction or EBSD in the AM steel (Figure 9b) and a fully martensitic lath microstructure was obtained. Nevertheless, the final microstructure of the AM steel was finer than in the as-built state and noticeably finer the microstructure of the conventional VACO 180 steel (Figure 11) in as-delivered condition. Additionally, the refinement of the AM steel can be contributed to the relatively low annealing temperature and short hold, which did not result in significant growth of newly formed recrystallised grains. On the other hand, VACO 180 steel was already delivered after 1 h annealing at 820 °C. However, shorter, 20 min annealing at the same temperature during solution annealing treatment of the hybrid part resulted in smaller prior austenite grain size and therefore also in a finer final microstructure. In this case of solution annealed hybrid part, the heat-affected layer at the interface was approximately 110 μm thick (Figure 7).

Micrographs showing a general view of the interface (Figure 7a–c) indicate that all three hybrid parts were of good quality, without large pores or other metallurgical defects. A few fine circular pores of approximately 1 μm and similarly sized particles were dispersed several layers from the interface. No metallurgical defects or lack of fusion were found directly at the interface.

### 3.3. Mechanical Properties

#### 3.3.1. Hardness

**Figure 12:**
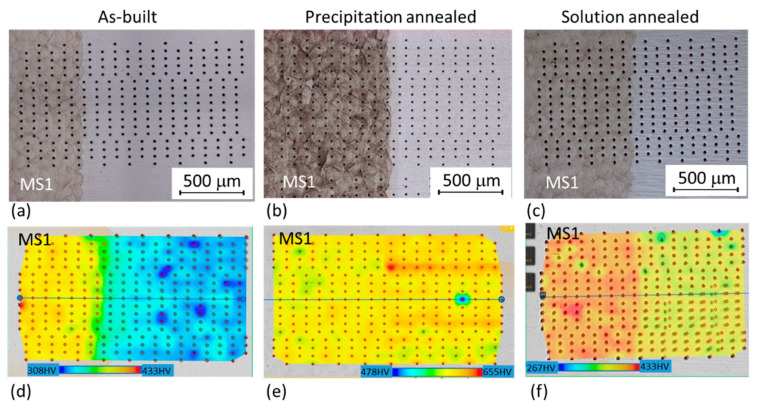
Microhardness mapping: (**a**,**d**) as-built; (**b**,**e**) precipitation annealed; and (**c**,**f**) solution annealed hybrid parts; (**a**–**c**) micrographs of the tested areas; and (**d**–**f**) corresponding microhardness maps across the joint between the AM and conventional steels.

**Figure 13:**
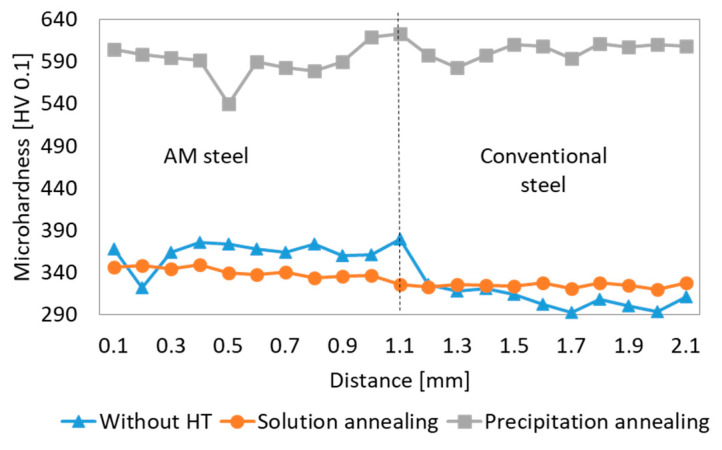
Microhardness across the joint between the AM and conventional steel. Vertical dotted line marks the interface.

Hardness mapping covering areas of approximately 1750 μm × 1000 μm was done across all three hybrid joints using HV 0.1 (Figure 12). The different hardness of the AM and conventional steel parts can be seen in the as-built hybrid sample and a small difference is also apparent in the solution annealed hybrid part. Almost the same hardness for both materials was found after precipitation annealing. Absolute hardness values around the interface were difficult to estimate from the maps, so hardness profiles were also measured (Figure 13). Hardness profiles were measured in all the hybrid parts from the AM side towards the conventional steel side. The interface is located at 1.1 mm on the horizontal axis (Figure 13). When evaluating the microhardness profiles of the hybrid parts, it should be considered that the microhardness of the AM steel could generally have a relatively large scatter within a single sample, depending on the position of the imprints. Various metallurgical defects, such as pores or inclusions, placed just below the measured surface can also result in an abrupt local drop of microhardness (as seen for example at 0.2 mm distance for the sample without heat treatment (HT) in Figure 13. This was confirmed by a reference microhardness measurement in AM steel without post-processing heat treatment. In total, ten measurements were carried out about 15 mm from the interface. Measured values were in the range of 355–426 HV 0.1, with the scatter of 71 points of HV 0.1.

There are small differences between the two sides of the interface. Particularly, in the specimens without post-processing heat treatment, the hardness of the conventional steel was significantly lower than the hardness in the AM steel. This is caused by the much finer microstructure of the matrix of AM steel, which was apparent in SEM micrographs and also EBSD images (Figure 7 and Figure 10). Moreover, this hardness difference in AM and conventional steels was also attributed to the high residual stresses produced in AM parts [29,30]. While the individual peak at 1.1 mm distance of hardness profile might have been contributed to a random local hardness variation, the hardness map in Figure 12a clearly confirms that the heat affected zone at the side of conventionally produced VACO 180 reached higher hardness (green areas) than the base material (blue area). This hardness change corresponds to the results observed in laser welds of the maraging steel [31], where the hardening of heat affected zone was explained by a precipitation process occurring during short-term heating. Such precipitation might contribute to the hardness increase in the hybrid part as well, as the surface of the conventional steel was heat-treated during the deposition of the first layers of AM steel. Nevertheless, this strengthening mechanism is not supported by SEM evidence as the early stages of the precipitation process do not have to be visible in SEM micrographs. On the other hand, the microstructure analysis confirmed the significant grain refinement of the surface of conventional steel in the heat affected zone (Figure 10a,b), which would contribute to the increased hardness of this area. This change of the grain size also proves that the surface temperature of the conventional steel during the deposition of AM steel was high enough to enable austenitic transformation.

Solution annealing post-processing treatment of the hybrid part changed the mechanical properties of the materials in different ways. While the hardness of the AM steel slightly decreased (due to the more uniform distribution of the alloying elements and the dissolution of the fine cell substructure), the hardness of the conventional steel VACO 180 increased slightly. Additionally, this hardness increase in VACO 180 is connected with a slight grain refinement caused by a shorter 20 min annealing hold compared to 60 min hold originally applied to the steel by the supplier. The microstructure changes equalised the hardness across the whole of the hybrid part, with only a slight reduction on the conventional steel side (Figure 13).

The hardness of both materials increased to 600 HV 0.1 after post-processing precipitation hardening. For both steels, AM and the conventionally prepared one, the same 6 h hold at 490 °C is recommended to gain the highest hardening effect obtained by intensive precipitation of Ni_3_Ti-based particles. The average hardness of the AM steel was several points lower than that of the conventional steel.

#### 3.3.2. Tensile Test

**Figure 14:**
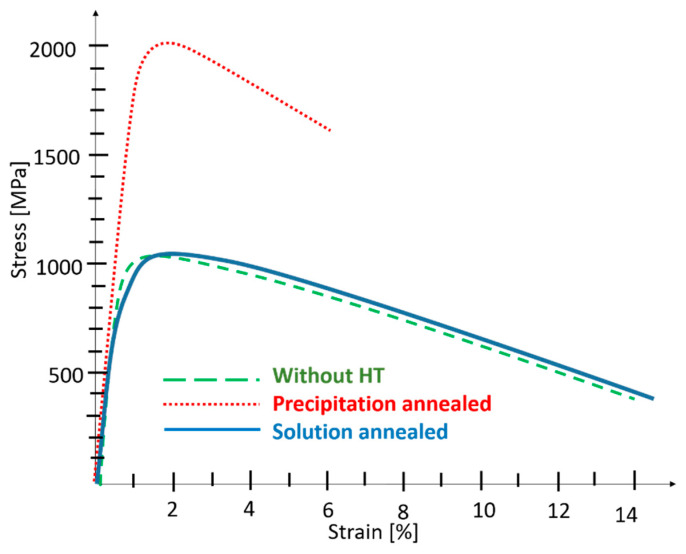
Representative stress-strain curves for the specimens from the hybrid parts. HT, heat treatment.

The tensile strength of the hybrid part in the as-built condition was 1029 MPa, with a total elongation of 14% (Figure 5 and Figure 14, Table 3). These values are just between those obtained for the AM and conventional steels. The yield and ultimate tensile strengths of the hybrid part were lower than for the as-built maraging steel. However, the yield strength is significantly higher (by approximately 150 MPa) than for the conventional steel. The ultimate tensile strength of the hybrid part is equal to that of the conventional steel. Total elongation shows the opposite trend; it was higher than in the AM steel but lower than in conventional steel. The as-built AM steel side showed markedly higher yield and ultimate tensile strengths than the as-received conventional steel and lower total elongation. This is a typical feature of AM maraging steel and the increased strength could be explained by the higher residual stresses and the very fine cellular microstructure created by high heating and cooling rates during the AM process. This processing conditions also resulted in high dislocation density within the AM steel which affects the strength. Moreover, the residual stresses and the presence of printing defects would be mainly responsible for the lower elongation of the AM steel. The fracture initiation on printing defects was previously confirmed by in situ tensile experiments even for the AM steel with porosity below 1% [13]. When comparing the mechanical properties of the AM and the conventionally produced sides of this hybrid part, one should also keep in mind that the as-received conventional steel had already been solution-annealed for one hour at 820 °C by the supplier. The properties of the AM steel correspond to the data sheet specifications for the build in the z-axis, except for HV 10, which is eight points higher than the specification (Table 3 and Table 4).

The precipitation-annealed hybrid part had the highest ultimate tensile strength, 2011 MPa, and a yield strength of 1945 MPa. The total elongation of the hybrid part was a mere 5%. The pattern of the properties of the hybrid part and the individual steels was the same as in the previous samples. Any heat treatment of AM steels generally shifts their tensile properties closer to the properties of the corresponding conventionally produced steel [17]. This holds for the VACO 180 and MS1 samples, which had very similar yield and ultimate tensile strengths after precipitation hardening. Still, there is a significant difference in the total elongation levels, with conventional steel reaching 9% and AM steel only 4%. It should be noted that 4% total elongation corresponds to the datasheet specifications for AM maraging steel along the z-axis (Table 4). Lower elongation is a general problem of AM steels generally attributed to the presence of printing defects rather than being related to any workplace or material-related issues. The mechanical properties of the steels were reflected in the properties of the hybrid part, whose yield and ultimate strengths were just slightly below those of both steels, whereas total elongation was just above the elongation of the AM steel. High hardness, around 600 HV 10, was obtained for both steels, which is in agreement with their datasheets. The increases in strength and hardness resulted from intensive precipitation of fine intermetallic particles in both steels, which contributed to the precipitation hardening effect, due to the interaction of the particles with dislocations during the straining. It was previously confirmed by atom probe tomography studies that the precipitates produced at peak hardening conditions in AM steel and conventional maraging steel had the same size, chemical composition, density and morphology [33]. Precipitation hardening reaches a peak value for this maraging steel after annealing at temperatures of 450–500 °C [16] which is the reason why heat treatment at 490 °C is recommended by the powder supplier. A fine dispersion of numerous Ni_3_Ti—type particles appears at those temperatures and only a negligible fraction of reversed austenite is formed at the same time. The total amount of austenite in the AM steel would be for peak annealing temperatures slightly higher than that in the conventional steel [28], due to the original 3–6% of retained austenite which remains in the microstructure of AM steel during the precipitation annealing. This combination of microstructure features maximises the strengthening of the steel, while coarsening of the precipitates and increase of reversed austenite fraction at higher annealing temperatures (above 500 °C) would result in a decline of strength and hardness after annealing at higher temperatures [28].

Solution annealing of the hybrid part resulted in the highest product of ultimate tensile strength and total elongation which could also be used as an approximate comparative measure of toughness. Both yield and tensile strengths are above the strengths of conventional steel. As already mentioned, conventional steel VACO 180 was received in a solution annealed condition. The post-processing heat treatment of the hybrid part was, therefore, the second solution-annealing operation carried out on the material. However, it had only a small effect on the mechanical properties. The hardness did not change at all and the yield and ultimate strength decreased insignificantly. On the other hand, solution annealing reduced the yield strength of AM steel by 100 MPa, its hardness by 23 points, and increased the total elongation by 3%, bringing its properties closer to the mechanical properties of conventional steel. This change of mechanical properties of the AM steel can be explained by the markedly changed microstructure on one hand and releasing of residual stresses on the other hand. Solution annealing caused a more homogeneous distribution of alloying elements connected with the dissolution of the original very fine cells. Recrystallisation of the as-built microstructure occurred while relatively low annealing temperature and short annealing time ensured very fine final grain size without any coarsening effect. This grain refinement is mainly responsible for maintaining still a quite high yield and tensile strength of the AM steel in comparison with coarser conventionally produced steel. The full austenitisation and subsequent slow cooling also resulted in the complete disappearance of the retained austenite. The fine, more homogeneous microstructure of AM steel without residual stresses achieved better total elongation of 15%, which could be the comparable value to the 16% elongation of conventional steel. As the solution annealing did not decrease the number of printing defects in the steel [28], the improvement of the elongation has to be attributed to the changes in the microstructure and residual stresses. After solution annealing, the yield tensile strength of the AM steel was observed to decrease more sharply than the ultimate tensile strength, increasing the work hardening rate of the AM steel. This could be caused by the change in the initial dislocation arrangement and density during the solution annealing of the AM steel. The high local dislocation density was already in the AM part before the mechanical testing [28], while annealing would result in recovery and recrystallisation.

For all conditions of the materials, the mechanical properties of MS1 and VACO 180 matched the values in their respective data sheets, as given in Table 4.

#### 3.3.3. Fracture Analysis

**Figure 15:**
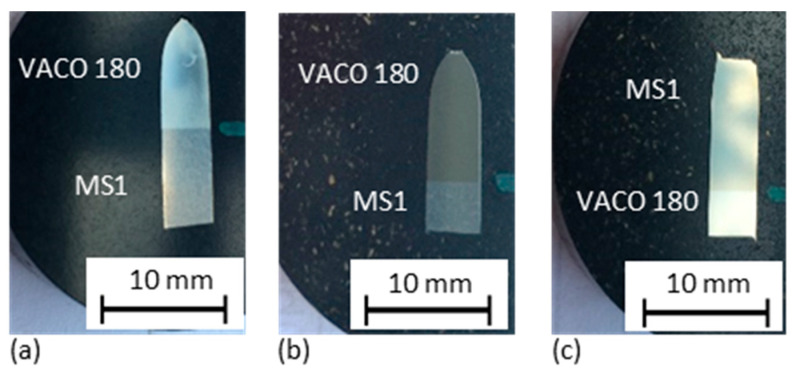
Metallographic longitudinal sections through fractured bars (the fracture area is at the top), showing the distance of the fracture from the MS1/VACO 180 interface for hybrid parts: (**a**) without heat treatment; (**b**) in the solution annealed condition; and (**c**) precipitation annealed conditions.

**Figure 16:**
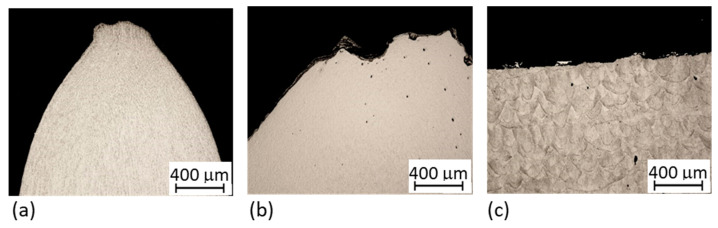
Detail of metallographic sections from the fracture region of hybrid parts: (**a**) without heat treatment; (**b**) solution annealed; (**c**) precipitation annealed. It was only in the precipitation annealed part that the fracture occurred in the additively manufactured steel.

**Figure 17:**
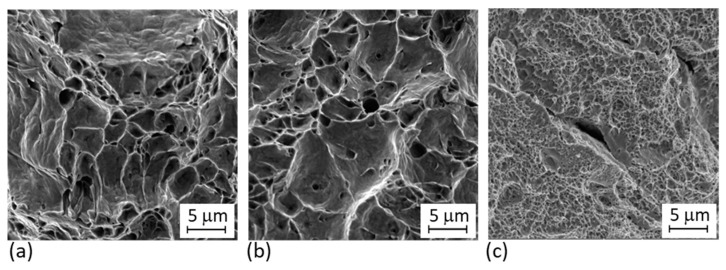
Fracture surfaces of hybrid parts: (**a**) without heat treatment; (**b**) solution annealed; (**c**) precipitation annealed.

Longitudinal metallographic sections of fractured tensile samples (Figure 15 and Figure 16) show that all the hybrid parts fractured far from the interface region. In the as-built (Figure 15a) and solution annealed (Figure 15b) hybrid samples, fracturing occurred in the conventional VACO 180 side. Large plastic deformation was localised at the fracture in the as-built and solution annealed hybrid part. In contrast, very little plastic deformation occurred around the fracture in the precipitation annealed part (Figure 15c). The precipitation annealed sample was the only one which fractured in the AM steel section. This may be due to the lower ductility in the AM portion than in the conventional steel section. These results are in agreement with findings from the fracture surface analysis (Figure 17). Hybrid samples in the as-built and solution annealed conditions had ductile fractures with a dimple morphology (Figure 15a,b). In both samples, the dimples were of various sizes. Small pores were found at the bottoms of the larger dimples in the solution annealed sample (Figure 15b). The fracture surface in the precipitation annealed part (Figure 15c) displays brittle fracture characteristics with a shallow relief and secondary cracks.

These results differ from the reported behaviour of similar and dissimilar hybrid joints of MS1, which typically fractured at the interface. This was the case with the MS1-H13 part [7,8], and the MS1-C300 part, where the fracture occurred in the AM side of the hybrid parts [8]. The authors contributed these fractures to the rapidly changing microstructures at the interface of similar joints [8], and the changing microstructure and chemical composition at the dissimilar joints interface [7]. The microstructure gradient in the specimens in our research is similar to the one in [8], which indicates that this need not be a critical issue if high-quality bonding between the materials is achieved. Previous work by the same authors involving dissimilar joints of MS1 and low alloy transformation induced plasticity (TRIP) 0.2C-1.5Mn-1.5Al-0.06Nb steel [6] also resulted in fractures in the base material several millimetres from the interface, despite significant microstructural and chemical gradients across the interface. This would again support the theory that high metallurgical purity of the interface area and good bonding of the deposited steel and the conventional substrate are in fact the main factors governing fracture location in hybrid parts.

## 4. Conclusions

Hybrid parts were successfully prepared using the DMLS method to deposit 18Ni300 maraging steel (MS1) on cylindrical semi-products of conventionally manufactured maraging steel (VACO 180). The hybrid parts were then analysed in three conditions: as-built, precipitation annealed at 490 °C and solution annealed at 820 °C. The mechanical properties of the hybrid parts were measured and compared to the mechanical properties of additively manufactured MS1 steel and VACO 180 steel alone. In all cases, the mechanical properties of the hybrid parts were similar to the mechanical properties of the individual steels and those specified in their datasheets.

The hybrid parts in the as-built condition had a yield strength of 947 MPa, an ultimate tensile strength of 1029 MPa and a total elongation of 14%. After post-build precipitation hardening, the hybrid parts showed a yield strength of 1943 MPa, an ultimate tensile strength of 2011 MPa and a total elongation of 5%. After solution annealing, there was a lower yield strength of 821 MPa and a tensile strength of 1043 MPa, accompanied by a higher total elongation of 14%. In the mechanical tests, the fracture always occurred in the base materials, several millimetres from the interface, which proves the high quality of the joint.

## Figures and Tables

**Table 1:** Chemical composition of the steels, including the values declared in the datasheet, in weight %. Composition of MS1 powder provided by EOS GmbH, composition of VACO 180 by Bohdan Bolzano, s.r.o.

Material	C	Cr	Mo	Ni	Co	Ti	Al
Data sheet	≤0.03	≤0.5	4.5–5.2	17–19	8.5–9.5	0.6–0.8	0.05–0.15
MS1	0.001	-	4.9	17.7	8.7	0.8	-
VACO 180	0.003	0.12	4.8	18.2	8.8	0.8	0.06

**Table 2:** Additive manufacturing parameters recommended by EOS GmbH.

Scanning Rate (mm/s)	Laser Power (W)	Layer Thickness (mm)	Misorientation Angle (°)	Hatch Spacing (μm)
960	285	0.04	67	110

**Table 3:** Mechanical properties of hybrid parts and individual steels determined by testing. Yield strength (YS), ultimate tensile strength (UTS), total elongation (EL), and hardness according to Vickers scale (HV 10). Average values and scatters of YS, UTS and EL are determined from 3 samples, HV 10 from 5 measurements.

Heat Treatment	Sample	YS [MPa]	UTS [MPa]	EL [%]	HV 10
Without heat treatment	Hybrid part	974 ± 8	1029 ± 2	14 ± 0	-
	VACO 180	820 ± 4	1030 ± 1	17 ± 1	312 ± 2
	MS1 as-built	1067 ± 15	1150 ± 10	12 ± 1	371 ± 2
Precipitation annealed	Hybrid part	1943 ± 6	2011 ± 3	5 ± 0	-
	VACO 180	1945 ± 13	2023 ± 13	9 ± 1	596 ± 6
	MS1	1958 ± 10	2015 ± 10	4 ± 1	601 ± 2
Solution annealed	Hybrid part	821 ± 22	1043 ± 1	14 ± 0	-
	VACO 180	816 ± 4	1022 ± 2	16 ± 1	312 ± 2
	MS1	879 ± 20	1120 ± 0	15 ± 1	338 ± 2

**Table 4:** Mechanical properties of steel 1.2709 according to datasheets, given for tensile testing according to ISO 6892-1:2009 (B) Annex D, proportional test pieces: diameter of neck area 5 mm, original gauge length 25 mm [32]. Yield strength (YS), ultimate tensile strength (UTS), total elongation (EL), hardness according to Vickers scale (HV). The properties are given for AM samples printed with the axis parallel to building direction (z). Precipitation annealed (PA), solution annealed (SA). Minimal values (Min.) are defined for Conventional steel.

Material	YS [MPa]	UTS [MPa]	EL [%]	HV 10
3D printed (z)	1000 ± 100	1100 ± 100	10 ± 4	327–363
3D printed (z) + PA	1990 ± 100	2050 ± 100	4 ± 2	513–612
3D printed (z) + SA	Not provided			
Conventional—PA	Min. 1910	Min. 1960	Min. 6–7	>570
Conventional—SA	Min. 640	930–1100	Min. 12	<350

## Data Availability

The raw/processed data required to reproduce these findings cannot be shared at this time as the data also forms part of an ongoing study.
